# Modeling Dynamics of the Cardiovascular System Using Fluid-Structure Interaction Methods

**DOI:** 10.3390/biology12071026

**Published:** 2023-07-21

**Authors:** Faiz Syed, Sahar Khan, Milan Toma

**Affiliations:** College of Osteopathic Medicine, New York Institute of Technology, Northern Boulevard, Old Westbury, NY 11568, USA; fsyed09@nyit.edu (F.S.); skhan87@nyit.edu (S.K.)

**Keywords:** fluid-structure interaction, fluid, structure, interaction, blood, flow, simulations, computational, circulation, modeling

## Abstract

**Simple Summary:**

Fluid-structure interaction algorithms are utilized to examine how the human circulatory system functions by simulating blood flow and capturing mechanical responses within blood vessels. These sophisticated algorithms take into account interactions between fluid dynamics, vessel walls, heart walls, and valves. By combining advanced medical imaging techniques with fluid-structure interaction models, it becomes possible to customize these models for individual patients. This customization enables clinicians to create personalized treatment plans. In this comprehensive article review, we explore various applications of fluid-structure interaction models in studying the cardiovascular system.

**Abstract:**

Using fluid-structure interaction algorithms to simulate the human circulatory system is an innovative approach that can provide valuable insights into cardiovascular dynamics. Fluid-structure interaction algorithms enable us to couple simulations of blood flow and mechanical responses of the blood vessels while taking into account interactions between fluid dynamics and structural behaviors of vessel walls, heart walls, or valves. In the context of the human circulatory system, these algorithms offer a more comprehensive representation by considering the complex interplay between blood flow and the elasticity of blood vessels. Algorithms that simulate fluid flow dynamics and the resulting forces exerted on vessel walls can capture phenomena such as wall deformation, arterial compliance, and the propagation of pressure waves throughout the cardiovascular system. These models enhance the understanding of vasculature properties in human anatomy. The utilization of fluid-structure interaction methods in combination with medical imaging can generate patient-specific models for individual patients to facilitate the process of devising treatment plans. This review evaluates current applications and implications of fluid-structure interaction algorithms with respect to the vasculature, while considering their potential role as a guidance tool for intervention procedures.

## 1. Introduction

**Figure 1:**
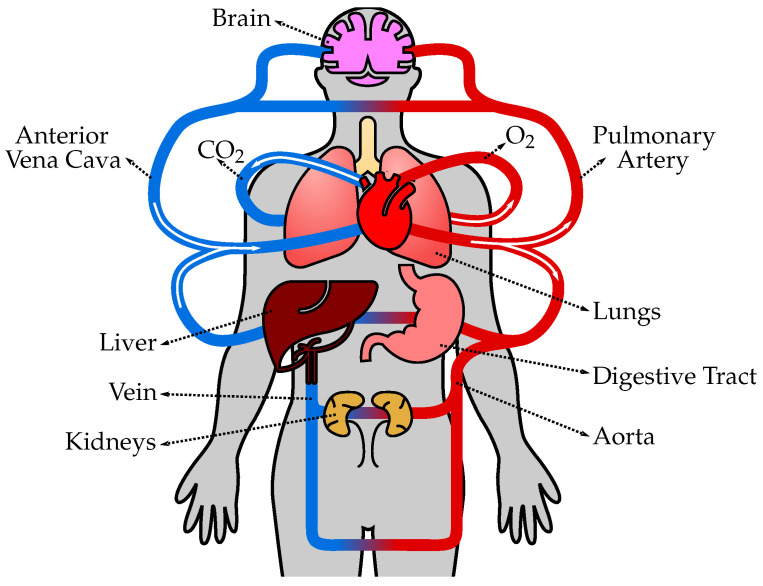
The human circulatory system consists of the heart, blood vessels, and blood, working together to transport oxygen, nutrients, hormones, and waste products throughout the body. The heart pumps oxygenated blood from the lungs to the body tissues through arteries, and deoxygenated blood is then returned to the heart through veins to be pumped to the lungs for oxygenation again.

The human circulatory system, also known as the cardiovascular system, is a complex network of blood vessels, organs, and tissues that transports oxygen, nutrients, hormones, and other essential substances throughout the body (Figure 1). It plays a crucial role in maintaining homeostasis and supporting the overall functioning of various organ systems. The circulatory system consists of several types of vessels that are responsible for different functions, as follows. (1) Arteries are blood vessels that carry oxygenated blood away from the heart and distribute it to various parts of the body. They have thick, elastic walls composed of smooth muscle and connective tissue, which allows them to withstand high blood pressure and pulsatile flow. Arteries progressively branch into smaller vessels called arterioles. (2) Arterioles are smaller branches of arteries that further divide into capillaries. They regulate blood flow and control blood pressure by constricting or dilating their smooth muscle walls. The diameter of arterioles can be adjusted to redirect blood flow to specific tissues or organs as needed. (3) Capillaries are the smallest and thinnest blood vessels in the circulatory system. They connect arterioles and venules and form an extensive network throughout the body’s tissues. Capillaries are responsible for the exchange of oxygen, nutrients, waste products, and hormones between the blood and surrounding tissues. The thin walls of capillaries allow for efficient diffusion of substances. (4) Venules are small vessels that receive deoxygenated blood from capillaries and merge to form veins. They collect blood from capillary beds and transport it to larger veins. Venules also play a role in the immune response by allowing white blood cells to migrate from the bloodstream to infected or injured tissues. (5) Veins are blood vessels that carry deoxygenated blood from various tissues and organs back to the heart. Unlike arteries, veins have thinner walls and lower blood pressure. They have valves that prevent the backflow of blood and aid in propelling blood toward the heart. Veins progressively merge to form larger vessels, returning blood to the heart through the superior and inferior venae cavae, and entering the right atrium. (6) The heart is a muscular organ located in the chest cavity and serves as the central pump of the circulatory system. It consists of four chambers: two atria (left and right) and two ventricles (left and right). The heart’s contraction (systole) and relaxation (diastole) generate the pumping action that propels blood throughout the circulatory system. The left side of the heart receives oxygenated blood from the lungs and pumps it into systemic circulation, while the right side receives deoxygenated blood from the body and pumps it to the lungs for oxygenation. (7) Pulmonary circulation refers to the pathway of blood from the heart to the lungs and back. Deoxygenated blood from the body enters the right atrium, passes through the right ventricle, and is pumped to the lungs via the pulmonary arteries. In the lungs, carbon dioxide is exchanged for oxygen, resulting in oxygenated blood. Oxygenated blood then returns to the heart through the pulmonary veins, entering the left atrium. (8) Systemic circulation is the pathway of blood from the heart to the body’s tissues and back. Oxygenated blood from the left atrium enters the left ventricle and is pumped out to the entire body through the aorta, the largest artery. Branches of the aorta distribute blood to various organs, and oxygen and nutrients are exchanged in the cap.

The prospective utilization of computational models to augment our comprehension regarding the circulatory system and hemodynamics is significant. Advanced imaging techniques such as computed tomography (CT) and magnetic resonance imaging (MRI) can be leveraged for the creation of three-dimensional models of blood vessels. Computational fluid dynamics, also known as CFD, constitutes one tool that facilitates analyzing the bloodstream through a vessel with tri-dimensional innovation geometry along with inlet–outlet velocity profiles that are extracted from non-invasive sources, like MRI scans detecting fluid movement [1]. The CFD has both strengths and weaknesses when it comes to modeling the circulatory system and hemodynamics. One of its major strengths is the ability to utilize noninvasive imaging techniques like MRI to track fluid movement [2]. However, there are also limitations associated with this approach, such as restrictions in resolution that may affect the accuracy of the results. Additionally, wall movements are not taken into consideration which can impact the overall accuracy of predictions made by these models. To overcome some of these limitations, fluid-structure interaction (FSI) algorithms prove advantageous as they allow for modeling deformable structures and interactions between fluids within those structures [3]. These algorithms enable a more comprehensive understanding of how fluid interacts with dynamic systems.

Nonetheless, the effectiveness of CFD is impeded by restricted resolution capabilities and an inability to consider wall movements associated with elastic artery walls during the computation of blood flow through these vessels, leading researchers to explore alternative approaches, such as the employment of FSI techniques. These methods effectively account for deformable structures and their interactions with the fluids, thereby surpassing the effectiveness and practicality achieved by conventional CFD. Furthermore, FSI methods enable a more realistic simulation of flow patterns and provide improved insight into how blood flow phenomena in vessels are affected overall [4,5].

The study of blood flow within the human circulatory system plays a vital role in understanding various cardiovascular diseases and designing effective treatment strategies. Computational modeling techniques have emerged as valuable tools for simulating blood flow dynamics. Among these techniques, FSI computational algorithms have gained considerable attention due to their ability to capture the complex interplay between fluid dynamics and deformable vessel walls. This literature review aims to explore the current state of research on the use of FSI computational algorithms to simulate blood flow in the human circulatory system, highlighting their applications, challenges, and potential clinical implications.

### 1.1. FSI Modeling Techniques

FSI modeling involves the simultaneous solution of fluid dynamics equations and structural mechanics equations to accurately capture the interaction between blood flow and the compliant vessel walls. Numerical methods, such as the finite element method (FEM) and finite volume method (FVM), are commonly employed to solve the coupled equations [6,7]. These techniques allow for the realistic simulation of phenomena such as wall deformation, fluid-structure interactions, and flow-induced forces, providing a comprehensive understanding of the hemodynamics within the circulatory system.

The immersed boundary method (IBM) is a technique for incorporating boundary conditions and collaborating with different solvers. It can be utilized in conjunction with both FEM and FVM, as well as other methods. The fluid component can be computed using the lattice Boltzmann methods, which have been proven to be highly effective compared to alternative approaches. The IBM has already been successfully employed in simulations of blood flow, exhibiting faster execution times than traditional CFD methods [8].

Alternatively, mesh-free approaches, also known as meshless methods, may be employed. These computational techniques do not require conventional structured or unstructured grids for discretizing the computing domain. Instead of using these traditional structures, distributed data points or particles are utilized to represent geometry and solve governing equations in a diverse manner. For blood vessel interactions applicable to FSI simulations, utilizing meshless methodologies can offer several advantages over other alternatives.

Key factors considered in FSI methods encompass wall shear stress, strain, stress, elastic modulus (E), and pulse wave velocity (PWV). Elevated levels of stress and strain on a blood vessel can increase the risk of complications such as rupture [9]. By utilizing FSI techniques to evaluate these parameters, it is possible to detect and manage potential issues at an early stage. Additionally, heightened aortic pulse wave velocity, which serves as a marker for arterial stiffness, has been associated with increased mortality rates in clinical populations [10]. Furthermore, employing FSI to measure the PWV of vessels enables us to assess their stiffness level and predict the likelihood of complications occurring [11].

### 1.2. Applications in Cardiovascular Research

FSI computational algorithms have been extensively utilized in cardiovascular research to investigate a wide range of physiological and pathological conditions. These include the assessment of hemodynamic parameters, such as wall shear stress, pressure distribution, and flow patterns, which are crucial for understanding disease progression and identifying regions of high stress and vulnerability. FSI models have been employed to study phenomena such as arterial stenosis, aneurysm formation and ruptures, atherosclerosis, and the performance of cardiovascular devices, such as stents and heart valves. The ability to simulate patient-specific geometries and conditions has opened up avenues for personalized medicine, allowing clinicians to tailor treatment strategies based on individual patient characteristics [12].

### 1.3. Challenges and Limitations

Despite their potential, FSI computational algorithms present certain challenges and limitations. Accurate and efficient modeling requires a realistic representation of both the fluid and structural domains, which demands high-fidelity imaging data and detailed constitutive models for vessel walls. The computational cost associated with solving the coupled equations is another challenge, especially for large-scale simulations and real-time clinical applications. The validation of FSI models against in vitro experiments or clinical data remains crucial to ensuring their reliability and clinical relevance. Additionally, the complexity of the circulatory system, including its pulsatile nature, multi-scale dynamics, and interactions with other organs, poses further challenges in accurately capturing the physiological behavior [13].

### 1.4. Clinical Implications

The use of FSI computational algorithms in simulating blood flow has the potential to significantly impact clinical practice. Patient-specific simulations can aid in preoperative planning, allowing surgeons to assess the effects of interventions on blood flow patterns and vessel mechanics. By providing insights into disease progression and rupture risk, FSI models can assist in the diagnosis and treatment of cardiovascular pathologies [14]. Furthermore, FSI simulations can guide the development and optimization of cardiovascular devices, leading to improved designs and enhanced patient outcomes. However, the translation of FSI modeling into routine clinical practices requires addressing technical challenges, standardizing methodologies, and integrating computational tools into existing healthcare systems.

## 2. Fluid-Structure Interaction Techniques

The FSI simulations involve solving a set of coupled partial differential equations that describe the fluid flow equations (e.g., Navier–Stokes equations) and the structural mechanics equations (e.g., the elasticity equations) [15]. These equations are solved iteratively to account for the mutual interaction between fluid and structure at each time step. The computational framework incorporates advanced numerical methods and algorithms to accurately capture the dynamic behavior of both fluid and structure [16]. In computational simulations involving FSI, several algorithms are employed to effectively model and capture the dynamic interaction between fluid flow and structural behavior, as follows.

– Monolithic approach: In the monolithic approach, the governing equations for fluid flow and structural mechanics are solved simultaneously within a single computational framework [17]. This approach involves coupling the fluid and structural equations and solving them together, often using an iterative procedure. It provides accurate and robust predictions of the fluid-structure interaction but can be computationally demanding.
– Partitioned approach: The partitioned approach separates the fluid and structural equations into two independent solvers, which are then coupled through an interface. Each solver operates independently, and the information is exchanged at the interface during the simulation. This approach allows for the use of existing specialized solvers for fluid and structural problems, making it computationally efficient [18].

The partitioned approach is further distinguished into two distinct strategies using computational techniques known as one-way and two-way methods. These approaches vary in their methodology towards simulating the interplay between fluid flow and solid structures.

– One-way FSI: In one-way FSI, also known as loosely coupled FSI, the fluid and structural domains are treated as separate and independent simulations. The fluid flow is first simulated using CFD techniques, and the resulting fluid forces are applied as boundary conditions to the structural analysis. The structural deformation, in turn, influences the fluid flow indirectly through these imposed boundary conditions. The information exchange between the fluid and structural domains is performed in a one-way manner, with the fluid providing the driving forces for the structural response. This approach is computationally efficient but assumes that the structural deformation has a negligible impact on the fluid flow [19].
– Two-way FSI: On the other hand, two-way FSI, also known as fully coupled FSI, considers the fluid and structure as a coupled system that interact with each other. In this approach, the fluid and structural equations are simultaneously solved in a fully coupled manner, accounting for the mutual influence between the two domains. The fluid forces affect the structural deformation, and the resulting deformation alters the fluid flow patterns. The information exchange occurs bidirectionally, allowing for a more accurate representation of the fluid-structure interaction. Two-way FSI is more computationally demanding but provides a more realistic representation of the physical phenomena involved [20].

The main difference between one-way and two-way FSI lies in the level of coupling and information exchange between the fluid and structural domains. One-way FSI assumes a one-sided influence of the fluid on the structure, while two-way FSI considers a fully coupled interaction where both the fluid and structure mutually affect each other. It is worth noting that the FSI algorithm choice depends on various factors, including the complexity of the problem, computational resources, desired accuracy, and the specific objectives of the simulation. Researchers and engineers select the most appropriate algorithm based on these considerations to ensure accurate and efficient simulations of fluid-structure interactions. The following are some commonly used FSI algorithms.

In the subsections below, the partitioned FSI approach is described using different methods. The coupling between the two solvers is achieved by explicitly defining boundary conditions at the fluid-structure interface, either through explicit iterations or other suitable strategies. A partitioned FSI approach is one of several methodologies used in FSI simulations. In contrast to the partitioned approach, some FSI techniques involve solving a monolithic system of equations, where the coupled equations for fluid and structure are solved simultaneously. In this monolithic approach, the fluid and structure equations are combined into a single set of equations, and a unified solver is used to solve them. The choice between the partitioned and monolithic approaches depends on various factors, such as the complexity of the problem, computational efficiency, and desired accuracy. However, the numerical methods presented in this manuscript specifically pertain to different approaches for solving the fluid dynamics component within the partitioned FSI framework. These methods offer researchers a range of options to tackle the fluid dynamics problem while adhering to the principles of partitioned coupling. By exploring and comparing these diverse numerical methods, researchers can gain insights into the strengths and limitations of each approach within the context of their FSI simulations.

### 2.1. Arbitrary Lagrangian–Eulerian Method

The interaction between fluid and its surrounding structures is an ever-present aspect of biology that plays an integral role in the vasculature of the human body [21]. Such interactions can be modeled through algorithms such as arbitrary Lagrangian–Eulerian (ALE) methods [22], which create a body-fitted resolution of the flow and stress distribution between fluid and its surrounding structure. The ALE methods are dependent upon the use of meshes with high density. As such, users tend to use simplified geometries to decrease the elements for stable calculations. They might, thus, be ill-suited to represent complex patient-specific geometries due to issues with modeling gaps between solid and fluid domains [23].

The ALE method combines the advantages of Lagrangian and Eulerian formulations by tracking the motion of the fluid mesh. It enables the mesh to move with the fluid, accommodating structural deformations, while also providing a fixed Eulerian framework for solving the fluid equations. This method is widely used in simulating FSI problems with large deformations. The ALE method can be implemented within both partitioned and monolithic approaches in FSI simulations, depending on the specific implementation and computational framework used.

– Partitioned approach: In the partitioned approach, the ALE method separates the fluid and structural equations into independent solvers. The fluid equations are solved using an Eulerian formulation, where the fluid mesh moves with the fluid motion. The structural equations are typically solved using a Lagrangian formulation, where the structural mesh remains fixed. The information is exchanged between the fluid and structural solvers at the fluid-structure interface, allowing for the interaction between the fluid and structure.
– Monolithic approach: In the monolithic approach, the ALE method involves solving the fluid and structural equations simultaneously within a single computational framework. The fluid and structural equations are coupled and solved together in a fully coupled manner. This approach provides a more tightly coupled representation of the fluid-structure interaction, where the motion of the fluid mesh and the structural deformation are considered concurrently.

Both approaches have their advantages and drawbacks. The partitioned approach allows for flexibility in choosing specialized solvers for fluid and structural problems, which can be computationally efficient. On the other hand, the monolithic approach provides a more integrated and accurate representation of the fluid-structure interaction but can be more computationally demanding. The choice between the partitioned and monolithic approaches depends on various factors, including the complexity of the problem, computational resources, desired accuracy, and the specific objectives of the simulation. Researchers and engineers select the most suitable approach based on these considerations to effectively capture the FSI using the ALE method.

### 2.2. Immersed Boundary Method

An alternative to body-fitted models is the immersed boundary formulation [24]. First introduced by Peskin in 1972, such methods use overlapping components of fluid and structure, avoiding many complications of mesh regeneration encountered in ALE methods [25]. One of the major advantages of the immersed method is that the equations are represented on a Cartesian grid, allowing for moving boundaries and simpler grid generation [23]. Thus, the IBM is commonly used when the fluid flow interacts with complex or deformable boundaries [26]. It represents the boundaries as an additional force term in the fluid equations, allowing for efficient simulations without explicitly modeling the boundary geometry. This method is particularly useful in simulating fluid-structure interactions with flexible structures, such as heart valves or blood vessel walls. The IBM method is typically implemented within the context of a partitioned approach in FSI simulations. In the partitioned approach, the fluid and structural equations are solved separately using independent solvers, and the information is exchanged at the fluid-structure interface. The fluid equations are solved using a traditional fluid solver, such as a finite volume or finite element method, while the structural equations are solved using a separate structural solver. The IBM represents the presence of the solid structure as an additional force term in the fluid equations, allowing for the interaction between the fluid and the deformable solid boundaries. The partitioned approach is well-suited for incorporating IBM because it allows for the decoupling of the fluid and structural solvers. The fluid solver does not need to explicitly know the details of the immersed boundaries, as the forces are incorporated as source terms in the fluid equations. This approach provides computational efficiency by leveraging existing specialized solvers for fluid and structural problems.

### 2.3. Embedded or Embedded-Boundary Method

The embedded or embedded-boundary method involves representing the solid structure as a mesh embedded within the fluid mesh. This method allows for efficient simulations by using the fluid mesh to solve the fluid equations and incorporating the solid boundary conditions within the embedded structure [27]. It is particularly useful when dealing with complex geometries or when the fluid-structure interface changes during the simulation. The embedded or embedded-boundary method can be implemented within the context of either a partitioned or monolithic algorithm in FSI simulations, depending on the specific implementation and computational framework used.

– Partitioned approach: In the partitioned approach, the embedded or embedded-boundary method involves separating the fluid and structural equations into independent solvers. The fluid equations are typically solved using a traditional fluid solver, such as a finite volume or finite element method, where the fluid mesh remains fixed. The structural equations are solved separately using a structural solver. The information is exchanged at the fluid-structure interface to account for the interaction between fluid and structure.
– Monolithic approach: In the monolithic approach, the embedded or embedded-boundary method solves the fluid and structural equations simultaneously within a single computational framework. The fluid and structural equations are coupled and solved together in a fully coupled manner. This approach provides a more tightly coupled representation of the fluid-structure interaction, where the fluid mesh and structural deformations are considered concurrently.

Both approaches have their advantages and limitations. The partitioned approach allows for flexibility in using specialized solvers for fluid and structural problems, which can be computationally efficient. The monolithic approach provides a more integrated and accurate representation of the fluid-structure interaction but may be more computationally demanding. The choice between the partitioned and monolithic approach for the embedded or embedded-boundary method depends on various factors, such as the specific problem, computational resources, desired accuracy, and the objectives of the simulation. Researchers and engineers select the most appropriate approach based on these considerations to effectively model and simulate the fluid-structure interaction using the embedded or embedded-boundary method. These algorithms can be further complemented with advanced numerical techniques, such as finite element methods, finite volume methods, or meshless methods, depending on the specific requirements and characteristics of the FSI problem.

### 2.4. Meshless Methods

Meshless methods, also known as mesh-free methods, are numerical techniques that do not rely on traditional structured or unstructured meshes for discretizing the computational domain [28]. Instead, these methods use a set of scattered data points or particles to represent the geometry and solve the governing equations. In the context of FSI simulations for blood vessel interactions, meshless methods offer several advantages.

One popular meshless method used in FSI simulations is the smoothed particle hydrodynamics (SPH) method. SPH represents the fluid and structure as a set of particles, where each particle carries properties such as position, velocity, and other relevant physical quantities. In SPH, the computational domain is discretized by placing particles throughout the domain, including the fluid and structural regions. For fluid simulation, SPH applies a smoothing kernel to evaluate the interactions between neighboring particles, allowing for the computation of fluid properties like pressure and velocity. The fluid equations, such as the Navier–Stokes equations, are solved by evaluating the interactions among the particles using this kernel function. The fluid particles interact with each other and with the solid particles representing the blood vessel walls, enabling the simulation of fluid-structure interactions.

In the case of blood vessel interaction, the deformations and motions of the vessel walls are typically governed by structural equations, such as the equations of linear elasticity. The solid particles representing the vessel walls are subject to external forces from the surrounding fluid particles, allowing for the simulation of the interaction between the fluid and the deformable vessel walls. Meshless methods in FSI simulations for blood vessel interaction offer several advantages. They can handle large deformations and complex geometries without the need for re-meshing or re-gridding. They are well-suited for modeling free surface flows and multiphase flows, where the fluid domain and interfaces can evolve dynamically. Meshless methods also provide flexibility in capturing fluid-structure interfaces and handling fluid-structure coupling. Ongoing research and advancements in meshless methods continue to improve their efficiency and accuracy in modeling FSI with complex geometries [29].

Similarly, meshless methods can be implemented using both partitioned and monolithic approaches in FSI simulations, depending on the specific implementation and computational framework used.

– Partitioned approach: In the partitioned approach, the meshless method separates the fluid and structural equations into independent solvers. The fluid equations are typically solved using the meshless method, where the computational domain is discretized by a set of particles or scattered data points. The structural equations are solved separately using another meshless method or a different numerical technique suitable for structural analysis. The information is exchanged at the fluid-structure interface to account for the interaction between fluid and structure.
– Monolithic approach: In the monolithic approach, the meshless method involves solving the fluid and structural equations simultaneously within a single computational framework. The fluid and structural equations are coupled and solved together in a fully coupled manner. This approach provides a more tightly coupled representation of the fluid-structure interaction, where the fluid particles and the structural particles or nodes interact directly and are solved concurrently.

The SPH technique has the inherent characteristic of preserving mass balance, and its ability to trace a surface automatically eliminates the need for repeated regeneration of mesh in response to deformations [30,31]. SPH discretizes a continuum into a set of particles, which interact through a kernel interpolation function with a radius known as the smoothing length. A particle’s physical properties are obtained by summing the properties of all of the particles within the kernel. The accuracy of SPH relies upon characteristics such as smoothing length, flow viscosity, and particle size amongst others [32,33]. The recent development of multi-resolution SPH further increases the resolution of fluid flow by splitting particles into regions of interest and merging particles where lower resolution is adequate [34,35]. One of the advantages of SPH is in modeling damage to blood vessels. Whereas mesh-based models are not well suited to model damage to an artery due to such damage continually decreasing stored energy, SPH can model such processes using particle-based methods to model the flow of blood. Each particle contains mechanical information such as stress, strain, position, and velocity. This allows for input equations to model stress on the surrounding structures and calculate the damage to the particles [36,37,38]. Due to these capabilities of modeling nonlinear large deformations, SPH has been used not just for the hemodynamics of arteries, but also for the more complex modeling of blood flow through the heart and its valves [39].

**Figure 2:**
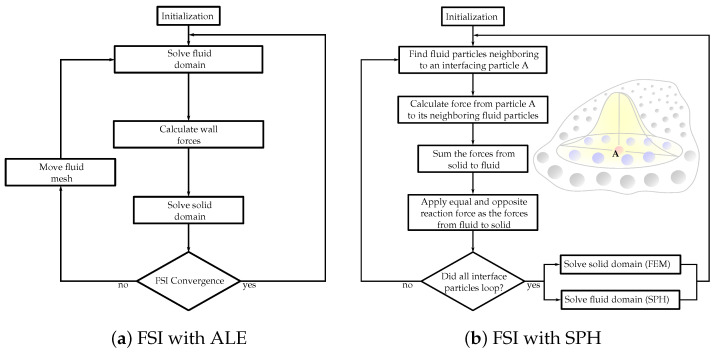
Flowcharts of the FSI solution algorithms with (**a**) Arbitrary Lagrangian–Eulerian (ALE) and (**b**) smoothed-particle hydrodynamics (SPH) methods.

The choice between the partitioned and monolithic approaches for meshless methods in FSI simulations depends on various factors, including the complexity of the problem, computational resources, desired accuracy, and the specific objectives of the simulation. Researchers and engineers select the most suitable approach based on these considerations to effectively capture the FSI using meshless methods. Figure 2 showcases the flowcharts demonstrating solution algorithms for FSI, incorporating both ALE and SPH methods.

### 2.5. Added Mass Effect

In FSI algorithms, the “added mass effect” refers to the phenomenon where the surrounding fluid exerts an inertial force on a structure undergoing motion, or, in other words, the surrounding fluid moves with the same velocity as that of the immersed boundary [40]. This effect occurs because the structure displaces fluid as it moves, resulting in an increase in mass that must be accelerated or decelerated along with the structure. In academic literature, the term “added mass effect” denotes numerical instabilities that often arise in the internal flow of an incompressible fluid having a density similar to or approaching that of surrounding structures. This phenomenon is generally not observed in problems pertaining to aeroelasticity due to relatively higher densities of solids compared to fluids. However, it carries noteworthy significance for diverse fields, including cardiovascular biomechanics, which deals with solids representing arteries engaging with blood as the fluid domain. The added mass effect is an important consideration in FSI simulations as it influences the dynamics and behavior of the coupled fluid-structure system.

**Figure 3:**
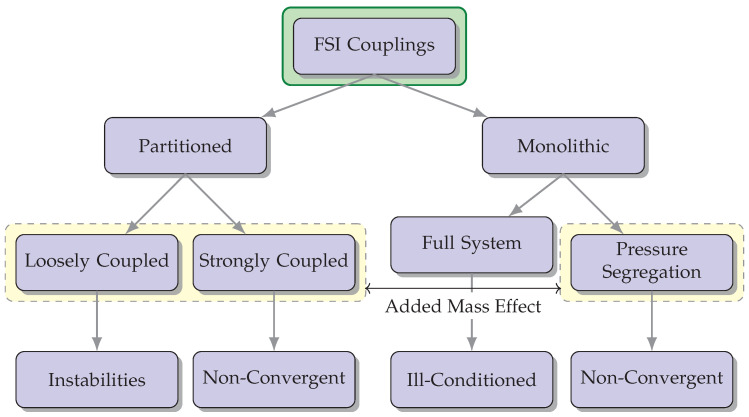
The diagram illustrates various possible combinations for FSI couplings and highlights potential concerns associated with each option. The incorporation of the “added mass effect” is a crucial factor to be taken into account during FSI simulations, as it has a significant impact on the dynamics and behavior of the interconnected fluid-structure system.

Strongly coupled methods necessitate achieving convergence of the fluid and solid variables at the boundary [41]. Despite requiring iterative procedures for convergence, they yield results that can be equally matched to non-partitioned strategies. Nevertheless, these techniques may encounter complications from an “added mass effect”, leading to a non-convergence of the solution. Consequently, specialized stabilization techniques have to be engineered with the intention of mitigating its impact and broadening their potential uses concerning FSI [42]. A compilation describing different feasible couplings for FSI is shown in Figure 3.

## 3. Blood Flow within Vascular Pathways

The arteries are intricate formations that show non-linear reactions to experiencing finite distortions. When creating models of such intricacy, multiple variables, including vessel elasticity and flow patterns, must be taken into account in FSI [43,44]. Patient-specific FSI studies are conducted by utilizing geometries specific to individual patients, which have been produced through CT or MRI imaging techniques [45,46,47,48]. This practice combines numerous two-dimensional image slices while also merging them together resulting in an accurate three-dimensional model unique to the patient being studied. As previously stated, but worth noting again, FSI simulations employ solvers that adhere to either the monolithic or partitioned approach. Under the latter method, fluid and structure are treated as distinct entities with their own respective mesh and equations. Transferring data between them requires an interface. Conversely, in the former method, both domains are integrated into a single unit along with a unified set of governing equations. Although this introduces additional computational complexity compared to its counterpart, it yields superior accuracy in results by comparison [49]. Advances in FSI open the door for its use in predictive diagnostics. Obtaining reliable measures of wall shear stress and hemodynamic risk indexes can support a clinician’s decision-making regarding the health of blood vessels within one’s body, as well as help guide treatment [50].

Cheng et al. challenged the classical Windkessel model by demonstrating that in addition to the heart’s power, the strain energy stored in deformed arterial vessels can contribute to propelling blood flow [51]. The study establishes a quantitative relationship between strain energy increment and functional and structural parameters of the aorta, such as blood pressure, stiffness, diameter, and wall thickness. By incorporating FSI, a mathematical model was developed to analyze the relationship between physiological parameters and blood supply to organs. The findings provide new insights into blood flow circulation, where cardiac output and arterial strain energy work together to distribute blood to distal organs and tissues. The model sheds light on the pathophysiological mechanisms of chronic diseases like cardio-cerebrovascular diseases and hypertension, aligning with epidemiological studies in these areas.

The predictive capabilities of a biomechanical model in evaluating the risks associated with a non-dilated thoracic aorta affected by Stanford type A dissection, a life-threatening condition, are assessed in [52]. A comprehensive FSI model was developed, considering realistic blood flow conditions, three-dimensional artery geometry, multiple artery layers, and in vivo-based physiological factors. The findings indicate that the wall shear stress is significantly reduced in the affected areas, potentially leading to aortic dilation and thrombus formation. The study also highlights the relationship between wall shear stress and blood flow patterns, with the aortic arch region near specific arteries being susceptible to rupture. These insights have clinical implications and the developed model can be customized for personalized assessments, serving as a predictive tool for estimating aneurysm growth and the risk of wall rupture in the thoracic aorta.

Azarnoosh et al. aimed to assess the impact of coarctation of the aorta (CoA) severity and duration on mechanical stimuli and arterial geometry changes using a rabbit model [53]. Different CoA severities and durations were induced, and FSI simulations were conducted. The results revealed vascular alterations, including thickening and stiffening, proximal to the coarctation, with increased severity and duration of CoA. FSI simulations showed a significant increase in wall tension with coarctation severity. Even mild CoA induced remodeling stimuli that surpassed values observed in adulthood, emphasizing the importance of early treatment. These findings provide insights into the mechanical stimuli associated with hypertension in patients with CoA and could aid in predicting the likelihood of hypertension in human patients.

The effect of uncertainty in the elastic module (E) on a patient-specific aortic model was investigated using an FSI approach [54]. The uncertainty quantification was performed using the generalized polynomial chaos (gPC) expansion technique. The results demonstrated the significance of the E parameter in the ascending aorta, while its impact on the descending tract was negligible. The study emphasized the importance of the image-based methodology in estimating E, enhancing the reliability of computational models for clinical use.

The accuracy of pulse wave imaging (PWI) in estimating PWV in stenotic vessels was assessed [55]. Consequently, computational FSI simulations and PWI in validation phantoms were used to evaluate the potential of PWI in assessing the mechanical properties of vulnerable plaques. The results demonstrated that PWI effectively distinguished the mechanical properties of plaque in phantoms, highlighting its potential for non-invasive estimation of plaque vulnerability and assessing stroke risk.

This study investigates the relationships between twelve multi-directional/topological wall shear stress (WSS) metrics; moreover, the formation of coronary plaques was investigated using both the CFD and dynamic FSI frameworks [56]. The results demonstrate the impact of stenosis percentage and lesion length on these metrics and highlight the influence of FSI simulations and coronary dynamics. The study also presents a patient case that explores the associations between the WSS metrics and changes in plaque morphology. These findings emphasize the potential role of coronary dynamics in altering WSS metrics and their implications for understanding coronary atherosclerosis. Further research is recommended to explore these relationships in different settings and longitudinal studies.

The role of biomechanics in coronary artery disease was investigated using FSI simulations to analyze patient-specific coronary biomechanics in both spatial and temporal domains [57]. A three-dimensional FSI model of the left anterior descending (LAD) coronary artery was developed, considering factors like myocardial bridging. Various biomechanical parameters including wall shear stress, strain, stress, and vessel fractional flow reserve (vFFR) were calculated across the cardiac cycle. The results highlighted regional and transient differences within the coronary artery and demonstrated the potential of FSI modeling to provide valuable insights for clinical evaluation and understanding of coronary pathology.

In the context of blood flow within vascular pathways, using FSI (as opposed to CFD) is significantly important because it enables a comprehensive understanding of the intricate relationship between the fluid dynamics of blood, the structural behavior of blood vessels, and the complexity of the entire circulatory system. It helps in predicting the impact of blood flow on the structural integrity of the vessels, such as the potential for wall deformation, vessel dilation, or the development of aneurysms. Furthermore, FSI simulations provide insights into hemodynamics, including the evaluation of factors such as blood velocity, pressure distribution, and shear forces within the vessels [58]. Understanding these parameters is essential for studying various cardiovascular conditions, such as atherosclerosis, hypertension, or the formation of blood clots. By considering the complex interplay between fluid dynamics and structural behavior, FSI analysis aids in advancing our understanding of circulatory system health and its associated pathologies. This knowledge contributes to the development of more accurate diagnostic tools, treatment strategies, and the design of interventions to enhance cardiovascular health.

## 4. Blood Flow within the Heart

Models of the heart have been generated using FSI accounting for precision in the opening and closing of valves with fluid flow throughout the heart chambers [59]. Such models mimic the native heart valves along with the dynamics that lead to the deterioration of valves, serving as a useful tool to streamline the process of designing valve prosthetics and replacements [60].

A study by Bornoff et al. focused on the use of a total artificial heart (TAH) as a bridge to transplant device for end-stage biventricular heart failure patients [61]. The Realheart TAH, a four-chamber artificial heart, mimics the native heart’s pumping technique using positive displacement and bileaflet mechanical heart valves. The researchers developed a method to simulate the hemodynamics of positive-displacement blood pumps using CFD and FSI. Simulations of the Realheart TAH under various conditions demonstrate accurate and robust performance, with good agreement between simulation and experimental results. The study provides valuable insights for future investigations and advancements in the field of TAH technology.

Cat et al. investigated the mechanics of calcified aortic valve stenosis (CAVS), a common cardiovascular disease characterized by calcium buildup and tissue thickening in the aortic valve [62]. Using a hybrid IBM-FEM, the researchers examine the dynamics and hemodynamic performance of the aortic valve under normal and calcified conditions. The results demonstrate that calcification significantly affects the valve’s elasticity, mobility, and opening area, leading to altered flow patterns and increased stress and strain. The computational model’s predicted hemodynamic parameters align with clinical risk classification, suggesting the potential for mathematical models to enhance our understanding of CAVS-induced ventricular dysfunction and support computational engineering-assisted medical diagnosis in aortic valve-related diseases.

Bileaflet mechanical heart valves (BMHVs) and the complications associated with their use, such as valve dysfunction, tissue overgrowth, hemolysis, and thromboembolism, were studied using FSI [63]. Thrombosis and thromboembolism are believed to be triggered by platelet activation caused by nonphysiological flow patterns and contact with foreign surfaces. The research highlights the significance of vorticity in platelet activation and aggregation in BMHV implants. A two-phase model incorporating blood and platelets, along with a fluid-structure interaction model, is employed to simulate the motion of the leaflets and estimate the platelet activation state (PAS). The study reveals that regions with higher vorticity fields contain platelets with higher PAS, indicating a correlation between vorticity and platelet activation. Furthermore, the research quantitatively demonstrates that areas with stronger vorticity exhibit higher platelet densities, suggesting that highly activated platelets aggregate in regions with increased vorticity.

A two-way immersed FSI computational model was developed and validated to study the impact of aortic annulus eccentricity and leaflet rigidity on the performance, thrombogenic risk, and calcification risk of bioprosthetic aortic valve replacements (BAVRs) [64]. The model demonstrates good agreement with experimental data and predicts that increasing eccentricities result in lower geometric orifice areas (GOAs) and higher transvalvular pressure gradients (TPGs), aligning with in vitro experiments. Higher eccentricities also indicate an increased risk of thrombus formation in the sinus of Valsalva. The study also examines the effect of leaflet rigidity and finds that more rigid leaflets lead to decreased valve performance, increased TPGs, and elevated risks of thrombus formation and valve calcification. This computational FSI model provides valuable insights into thrombogenic biomarkers and offers a novel tool for device manufacturers and clinical practitioners.

Patient-specific post-surgery type A aortic dissection (TAAD) models were created using computed tomography angiography images, and fully coupled two-way FSI simulations were conducted [5]. In this study, the impact of aortic wall compliance on intraluminal hemodynamics in surgically repaired TAAD was investigated. A comparison was made between FSI simulations, which accounted for aortic wall compliance and rigid wall simulations. The FSI model showed lower blood velocities and wall shear stress along the dissected aorta, resulting in larger areas exposed to low time-averaged wall shear stress. The FSI models also exhibited more disturbed flow with increased turbulence intensity compared to the rigid wall models. The pressure difference between the true and false lumen was minimally affected by wall compliance. Simulations with different Young’s moduli indicated that a more compliant wall further reduced velocity and wall shear stress due to increased displacements. This study highlights the significance of FSI simulations in accurately predicting low wall shear stress regions in surgically repaired TAAD, while rigid wall computational fluid dynamics simulations are sufficient for predicting luminal pressure differences.

Most FSI models simulate the mitral valve, aiming to enhance the detection of blood leakage and assess the quality of valve closure. An FSI model utilizes the immersed boundary method to create a contact map, accurately representing the closure of the mitral valve without the appearance of orifice holes [65]. The study addresses convergence issues and demonstrates the effectiveness of the method in three clinical scenarios: mitral valve with leakage, bulging, and healthy conditions. The contact map enables easy detection of leakage, identification of leakage sources, and evaluation of closure quality. The method improves simulation quality, offers a spatial assessment of valve closure, and provides comparable speed, the ability to handle large deformations, and detailed contact representation. Other FSI models employed mesh-free techniques to achieve similar objectives [66,67,68,69,70].

Fang et al. conducted an FSI analysis to calculate hemodynamic parameters and employed particle residual rate (PRR) and blood renewal rate (BRR) analyses to assess thrombogenesis dynamics [71]. This study focused on comparing the left atrial appendage (LAA) morphology and hemodynamics in patients with and without a history of stroke, who had non-valvular atrial fibrillation (NVAF). The LAA is a common source of cardioembolic strokes in NVAF patients. The researchers reconstructed LAA geometric models and measured morphological parameters. The results indicated that patients with a history of stroke had smaller LAA tortuosity and orifice areas, lower orifice velocities, but higher PRR and BRR values compared to those without a stroke history. These findings suggest that LAA morphology and hemodynamic parameters may serve as potential indicators for assessing stroke risk in NVAF patients.

The utilization of computational techniques can be a viable and economical approach for assessing the flow parameters associated with heart valves. The inherent difficulties relating to long-term stability and biocompatibility in valve replacement or repair have underscored the necessity for more resilient methods that could address valvular disease effectively [72]. A thorough analysis of engineering practices and computational methodologies utilized in investigating the dynamic behavior of healthy and diseased heart valve closures is available in reference [73].

In relation to the blood flow within the heart, FSI plays a crucial role as it facilitates an all-encompassing comprehension of the intricate relationship between blood fluid dynamics, heart wall structural mechanics, and valve performance. This is particularly significant in understanding blood flow mechanisms within the heart. The FSI analysis enables the investigation into how the flow of blood interacts with the flexible and dynamic nature of the heart valves, which play a crucial role in ensuring proper unidirectional blood flow through the different chambers of the heart. By considering the interaction between the fluid and the valve leaflets, FSI simulations can provide insights into valve dynamics, including the evaluation of valve function, assessment of regurgitation or stenosis, and prediction of potential complications related to valve performance. Consequently, FSI studies contribute to advancing our knowledge of cardiovascular health, aiding in the development of diagnostic tools, treatment strategies, and the design of more effective prosthetic heart valves.

## 5. Blood Flow within Aneurysms

Aneurysms can form anywhere within the arterial system, including the intracranial, aortic, abdominal, visceral, and peripheral arteries. Aneurysm diameter and expansion rate are risk factors that are assessed to estimate rupture risk. In cases in which the aneurysm is the result of thrombosis, diameter is also used to guide anticoagulant treatment. Computational models can be used to predict growth and likelihood of rupture of aneurysms based on parameters such as wall stress, oscillatory shear index, and patterns of blood flow [74].

Blood flow in the brain occurs at different scales from large arteries with diameters of greater than 0.5 mm to smaller arteries and arterioles that can be as narrow as 10 μm. Cerebral aneurysms can have devastating consequences, such as subarachnoid hemorrhage, which leaves 60% of those afflicted with it dead or disabled. Most cerebral aneurysms go unnoticed until they rupture, or are incidentally noticed by brain imaging for another condition [75]. Quantitative analyses using FSI are useful for modeling risk factors for aneurysms. A study using FSI modeling blood flow through the cerebral vasculature determined arterial bifurcations to be the most vulnerable locations for the development of intracranial aneurysms, an important observation that suggests that risk factors for cerebral aneurysms, such as atherosclerosis, are location dependent [76]. FSI has also been used to study hemodynamics, which could exacerbate a cerebral aneurysm. In a study modeling an aneurysm in the middle cerebral artery, FSI simulations demonstrated that a partially blocked middle cerebral artery resulted in greater stress at the aneurysm point with an increased likelihood of aneurysm ruptures as compared to a middle cerebral artery aneurysm, where the remainder of the vessel was patent [77]. Angiography images can be obtained to provide such information regarding wall stresses and the risk of aneurysm ruptures for individualized patients [78]. Individualized models based on patient-specific properties showed significant differences from a constant model, with the patient-specific model demonstrating significantly altered stress at the site of the aneurysm compared to the constant model [79,80]. Patient-specific models of the cerebral vasculature have made it possible to study the effects of changes in motion on the resulting hemodynamics relating to aneurysms. In a study using CT images to create a FSI model of the cerebral arteries, it was shown that traumatic brain injuries showed an increase in the pressure and velocity of the blood in cerebral arteries following a simulated traumatic brain injury, thus providing important information regarding the risk of aneurysm rupture with traumatic brain injury [81]. Patient-specific models have also been used for examining uncommon manifestations of aneurysms such as cranial nerve palsies. A study using FSI examined an aneurysm in a patient with facial nerve palsy, determining that the aneurysm had the highest stress in the region of the facial nerve, causing the patient’s symptoms [82].

FSI has also demonstrated its use in modeling aneurysms that occur within the aorta [83]. FSI models have shown that stress and potential rupture of an aneurysm of the abdominal aorta are dependent upon the shape and diameter of the aneurysm. Thus, such simulations may be important in determining the severity of the disease [84]. Simulation results indicate that wall shear stress increases in areas of aneurysms that impinge upon blood flow and decreases in areas distal to this site, an important development in monitoring the progression of an aneurysm, as from a mechanical perspective, aneurysm rupture occurs when wall stress exceeds arterial wall strength [85,86,87,88]. Aneurysm rupture risk is traditionally assessed by measuring the diameter of the aorta. However, rupture risk assessment is possible using biomechanical analyses taking into account parameters, such as mechanical stress, shear stress, and strain if boundary conditions are truly patient-specific. Patient-specific boundary conditions can be evaluated using a framework that individualizes inflow and outflow conditions, which can be obtained through phase contrast MRI images [89]. A study using FSI to compare the rupture risk of an abdominal aortic aneurysm between patients found that those with larger iliac artery bifurcations had greater wall shear stress, which reduced the likelihood of aneurysm ruptures. Abdominal aortic aneurysms have been shown to develop in regions with less wall shear stress [90].

Certain genetic conditions, such as Marfan syndrome, affect the elasticity of the aorta and predispose patients to aneurysms. FSI computations using a hyperelastic model of the aorta have compared the wall shear stress in Marfan syndrome patients as compared to controls and determined that wall shear stress was lower in those with Marfan syndrome, indicating vascular remodeling in the regions with the aneurysm [91]. A phenomenon known as “buckling” occurs in aneurysmal and normal arteries when the lumen pressure exceeds a critical buckling pressure. The critical buckling pressure is met when the artery becomes unstable due to quick increases in fluid deflections. Aneurysm formation reduces the axial tension on a vessel and decreases the critical buckling pressure, leading to the increased likelihood of mechanical instability, which could result in aneurysm ruptures [92]. Studies comparing the use of elastic against hyperelastic materials in FSI modeling the vasculature in the aorta show that the differences in materials result in a sizable difference between the two in aneurysm initiation and the stress response of aneurysms [93]. However, regardless of differences in elasticity or blood pressure, patient-specific analyses have shown that the difference between ruptured and unruptured aneurysms is higher von Mises stress and greater displacement at the dome [94].

The use of FSI modeling can further be incorporated into the treatment and management of aneurysms, as it can guide stent placement. FSI analysis was used to evaluate four different stents of varying materials, and factored in properties such as mechanical stresses, wall shear stress, and wall compliance [14]. Such an analysis can guide vascular surgeons in choosing the proper stent to use during surgical procedures. In another study, FSI was used to observe blood flow and wall shear stress through a nitinol stent graft placed in the aorta [95]. In these ways, FSI can aid in the optimization of stent placement and assessment of functionality.

A computational framework was developed to investigate and compare flow patterns in the aorta before and after thoracic aortic aneurysm (TAA) formation [96]. The framework utilized a deformable wall model and was validated through in vitro experiments considering patient-specific geometries and physiological conditions. Particle image velocimetry (PIV) was used to evaluate the complex flow behaviors in both pre-aneurysmal and post-aneurysmal aortas. The experimental results aligned with the numerical study, showing a small vortex near the aortic arch in the pre-aneurysmal aorta, which may contribute to aneurysm formation. The study also indicated a correlation between high endothelial cell action potential (ECAP) and recirculation regions, suggesting the possibility of thrombus development. This image-based FSI model, combined with in vitro experiments, has the potential for virtual implantation of stent grafts to treat TAA, providing valuable insights for future interventions.

FSI models have been used to compare and contrast flow velocity, pressure distribution, deformation, and wall stress of two different methods of aortic aneurysm repair: stenting and wrapping. Wrapping involves placing polytetrafluoroethylene (PTFE) material around the aneurysm. It has been found that stenting creates significant changes in blood flow patterns within the aneurysm, while wrapping increases the thickness of the aneurysm and the strength of the vessel wall. Moreover, wrapping was found to have a significantly reduced amount of von Mises stress compared to the stent models [97].

A tool combining measured aorta displacements and computed blood velocity field was developed to estimate blood flow-induced loads on the artery wall [98]. The SPH model was adapted for solving the 3D Navier–Stokes equations in a domain with prescribed motion. By comparing the results with different assumptions about the arterial wall’s motion, significant differences were observed in the distribution of wall shear stress, highlighting the importance of considering the moving arterial wall in the analysis.

In the context of aneurysm repair, FSI holds particular significance as it can contribute to the selection of appropriate treatment strategies [72]. By integrating patient-specific geometries, material properties, and hemodynamic conditions, FSI models enable clinicians and researchers to assess the performance and efficacy of different interventions, such as stent graft placement and surgical techniques. A comprehensive understanding of the FSI gained from these simulations can inform the decision-making process and ultimately lead to improved outcomes in aneurysm repair procedures.

## 6. Discussion

The utilization of FSI holds significant value in assessing and analyzing a diverse array of vessels within the human body. By considering the dynamic interaction between the fluid flow and the structural response of the vessel walls, FSI simulations provide insights into the complex biomechanical behavior of the cardiovascular system. By employing FSI algorithms, researchers and clinicians can unlock a deeper understanding of the complex hemodynamic characteristics of the circulatory system. FSI simulations provide a powerful means to explore the intricate interplay between blood flow and vessel mechanics, shedding light on crucial aspects such as blood pressure distribution and flow patterns. These simulations offer insights into the development and progression of cardiovascular diseases, unraveling the underlying mechanisms and identifying potential targets for intervention. Furthermore, FSI simulations play a vital role in the design and optimization of medical devices used in cardiovascular interventions [99]. By assessing device performance within the context of realistic physiological conditions, FSI simulations aid in refining device designs and improving their efficacy and safety. Additionally, FSI simulations have the capacity to investigate patient-specific scenarios, integrating personalized geometries and physiological properties into the models. This personalized approach allows for a tailored analysis of specific clinical cases, enhancing diagnostic capabilities and enabling more precise treatment planning. The use of FSI algorithms in simulating the human circulatory system holds great promise for advancing our understanding of cardiovascular dynamics and ultimately enhancing patient care. The seamless integration of fluid and structural mechanics within a single simulation framework empowers researchers and clinicians to delve into the intricate details of blood flow and vessel behavior, leading to improved cardiovascular diagnostics, innovative treatment strategies, and ultimately better patient outcomes.

Experimental validation of computational studies is crucial, especially when simulating processes within the human body, due to several reasons. First and foremost, the human body is a complex and intricate system with numerous variables, making it challenging to capture all aspects accurately in computational models [100]. Experimental validation provides an opportunity to compare model predictions with real-world observations, ensuring that the simulations capture the essential features and dynamics of the biological system. Additionally, experimental validation helps identify potential discrepancies or limitations in the computational models. It allows researchers to assess the accuracy, reliability, and predictive capability of the simulations. By comparing the results of computational models with experimental data, researchers can verify the assumptions, algorithms, and parameters used in the simulations. If inconsistencies arise, it may indicate the need for refinement or adjustment of the computational models to better reflect the real physiological processes [101]. Furthermore, experimental validation provides an essential means of building trust and confidence in computational models. It allows researchers and medical professionals to assess the validity and applicability of the models in clinical decision-making. When computational simulations are successfully validated against experimental data, they can provide valuable insights, support medical diagnoses, guide treatment strategies, and aid in the development of new therapies or interventions. Overall, experimental validation is essential for strengthening the reliability and practicality of computational studies conducted within the human body. Its primary purpose is to verify that simulations accurately depict the physiological mechanisms at work, thus enabling medical professionals and researchers to make sound judgments and progress in medicine [102]. However, many uncertainties enter the process in both the experimental and computational techniques. For example, the process of imaging subject-specific geometries involves both experimental and computational techniques, all of which introduce various uncertainties. It is crucial to account for these uncertainties as they can lead to erroneous computational analyses and geometric errors in the resulting model. In order to mitigate some sources of geometric error, preparation methods have been developed [103]. However, even with these methods, the resulting 3D geometry often does not accurately retain the original dimensions before excision from the body.

Numerical FSI methods for simulating blood flow within the human circulatory system can be enhanced by incorporating artificial intelligence (AI) techniques [104]. AI has the potential to revolutionize FSI simulations by improving their accuracy, efficiency, and predictive capabilities. Advanced machine learning algorithms can be trained on large datasets of simulated or experimental FSI data to learn complex relationships between input parameters and output variables. By leveraging this learned knowledge, AI algorithms can assist in predicting and optimizing blood flow patterns, identifying regions of high stress or risk, and improving the accuracy of FSI simulations. This can lead to better understanding and characterization of cardiovascular diseases, as well as more effective treatment planning. Furthermore, AI can contribute to the development of personalized FSI models by incorporating patient-specific data. By leveraging AI techniques, such as image recognition and pattern analysis, it is possible to extract detailed anatomical and physiological information from medical imaging data [105]. This information can then be integrated into FSI simulations to create patient-specific models, allowing for a more accurate and tailored analysis of individual cases.

## 7. Conclusions

The field of FSI offers a powerful quantitative approach to modeling patient-specific anatomy and the vasculature, enabling clinicians to gain detailed insights into the dynamics of blood flow within individual patients. Incorporating patient-specific geometries and physiological properties, FSI simulations provide a comprehensive view of how blood flows through the vasculature and the resulting forces acting on the vessel walls. This capability allows clinicians to identify regions of the vasculature that are subjected to varying forces, such as shear stress and von Mises stress, which are crucial indicators of vascular health. Through FSI modeling, clinicians can detect and analyze vascular disorders, including aneurysms, characterized by abnormal dilatation of blood vessels. By observing the effects of different types of stent treatments using FSI models, clinicians can make informed decisions on stent selection and placement, optimizing the restoration of blood flow and improving patient outcomes.

The use of FSI computational algorithms in simulating blood flow within the human circulatory system holds immense promise for advancing our understanding of cardiovascular diseases and enhancing clinical practice. The ability to capture the intricate interplay between fluid dynamics and vessel wall mechanics empowers researchers and clinicians to conduct detailed investigations of both physiological and pathological conditions. FSI simulations enable the study of complex phenomena, such as thrombosis, atherosclerosis, and hemodynamic abnormalities, facilitating a deeper understanding of disease mechanisms and potential treatment strategies. However, it is essential to address technical challenges associated with FSI modeling, including computational efficiency, accuracy, and model validation against experimental and clinical data.

This review has shed light on the current applications of FSI techniques in medical fields. By employing FSI, researchers and clinicians can explore a wide range of possibilities for improved diagnostics and treatments. However, it is essential to continuously assess the computational efficiency and accuracy of FSI techniques in comparison to experimental and clinical approaches, e.g., vFFR. Such a comparative analysis would provide a comprehensive understanding of the strengths and limitations of FSI. Furthermore, the path toward standardization emerges as a critical consideration. Standardization efforts are necessary to ensure consistent and reliable results as FSI techniques gain prominence. By discussing the current state of standardization, challenges faced, and potential strategies to achieve it, the review can contribute to the field’s advancement [102,106].

Hence, to fully realize the potential of FSI modeling, further research is necessary to refine and optimize the computational algorithms and numerical methods employed. Validation of FSI models against experimental and clinical data is crucial to ensure their reliability and accuracy in predicting real-world scenarios. Moreover, establishing guidelines for integrating FSI simulations into routine clinical practice is vital for effectively utilizing this technology for patient care [102]. With continued advancements and collaborative efforts between researchers, clinicians, and engineers, FSI modeling has the potential to revolutionize cardiovascular research, enabling the development of more personalized and effective diagnostic tools and treatment strategies tailored to individual patients.

## Data Availability

Not applicable.

## Abbreviations

The following abbreviations are used in this manuscript:

FSI	fluid-structure interaction
CT	computed tomography
MRI	magnetic resonance imaging
CFD	computational fluid dynamics
FEM	finite element method
FVM	finite volume method
IMB	immersed boundary method
ALE	arbitrary Lagrangian–Eulerian
SPH	smoothed particle hydrodynamics
CoA	coarctation of the aorta
E	elastic module
gPC	generalized polynomial Chaos
PWI	pulse wave imaging
PWV	pulse wave velocity
WSS	wall shear stress
LAD	left anterior descending
vFFR	vessel fractional flow reserve
TAH	total artificial heart
CAVS	calcified aortic valve stenosis
BMHV	bileaflet mechanical heart valve
PAS	platelet activation state
BAVR	bioprosthetic aortic valve replacement
GOA	geometric orifice area
TPG	transvalvular pressure gradient
TAAD	type A aortic dissection
PRR	particle residual rate
BRR	blood renewal rate
LAA	left atrial appendage
NVAF	non-valvular atrial fibrillation
TAA	thoracic aortic aneurysm
PIV	particle image velocimetry
ECAP	Endothelial cell action potential
PTFE	polytetrafluoroethylene
AI	artificial intelligence
